# Somatic mutations of *PREX2* gene in patients with hepatocellular carcinoma

**DOI:** 10.1038/s41598-018-36810-5

**Published:** 2019-02-22

**Authors:** Ming-Hui Yang, Chia-Hung Yen, Yen-Fu Chen, Cheng-Chieh Fang, Chung-Hsien Li, Kuo-Jui Lee, Yi-Hsiung Lin, Chien-Hui Weng, Tze-Tze Liu, Shiu-Feng Huang, Bin Tean Teh, Yi-Ming Arthur Chen

**Affiliations:** 1 Center for Infectious Disease and Cancer Research (CICAR), Kaohsiung Medical University, Kaohsiung, 80708 Taiwan; 2 Institute of Biological Chemistry, Academia Sinica, Taipei, 11529 Taiwan; 3 Master Program in Clinical Pharmacogenomics and Pharmacoproteomics, College of Pharmacy, Taipei Medical University, Taipei, 110 Taiwan; 4 Graduate Institute of Natural Products, College of Pharmacy, Kaohsiung Medical University, Kaohsiung, 80708 Taiwan; 5 VYM Genome Research Center, National Yang-Ming University, Taipei, 11221 Taiwan; 6 Institute of Molecular and Genomic Medicine, National Health Research Institutes, Miaoli, 35053 Taiwan; 7 Laboratory of Cancer Epigenome, Division of Medical Sciences, National Cancer Centre, Singapore, 169610 Singapore

## Abstract

Characterized with a high recurrence rate and low detection rate, prevention is the best approach to reduce mortality in hepatocellular carcinoma (HCC). The overexpression of Phosphatidylinositol-3,4,5-Trisphosphate Dependent Rac Exchange Factor 2 (PREX2) is observed in various tumors, including HCC; and the frequent PREX2 mutations in melanoma are associated with invasiveness. We sought to identify somatic mutations and the functional changes in mutational signatures of PREX2. Genomic DNA sequencing was performed in 68 HCC samples with three types of hepatitis viral infection status: HBs Ag-positive, anti-HCV Ab-positive, and negative for any hepatitis B or C markers. Stabilities and interactions of proteins as well as cell proliferation and migration were evaluated. Fourteen non-silent point mutations in *PREX2* were detected, with 16 of 68 HCC patients harboring at least one non-silent mutation. All mutant forms of PREX2, except for K400f, had an extended half-life compared with wild-type PREX2. Moreover, only the half-life of S1113R was twice that of the wild-type. PREX2 mutant-S1113R also promoted migration and activated the AKT pathway as well as impaired HectH9-mediated ubiquitination. Our study identified a gain-of-function mutation of PREX2 – S1113R in HCC. Such mutation enhanced PREX2 protein stability, promoted cell proliferation, and was associated with aggressiveness of HCC.

## Introduction

Hepatocellular carcinoma (HCC) is among the top ten most common types of newly diagnosed cancer worldwide and the second most common cause of cancer-related death. In less developed regions, the incidence of HCC among males is high, following only lung cancer^1^. Because the death rate approaches the incidence rate (a ratio of 0.95), prevention is the best approach to reduce HCC mortality^1^. In addition, elucidation of the somatic genetic alterations in HCC may be beneficial.

Phosphatidylinositol 3,4,5-trisphosphate-dependent Rac exchanger 2 (PREX2, also known as P-Rex2a) is a 182 kDa protein sensitive to PI-3-kinase (PI3K) belonging to the Rac guanine nucleotide exchange factor family (RacGEFs)^2^. PREX2 is capable of inhibiting the activity of phosphatase and tensin homolog (PTEN) and thus regulates the downstream PI3K signaling pathway^3^. As a result, PREX2 is considered to be an oncoprotein. Recently, overexpression of PREX2 has been observed in various tumors, including glioma, breast, ovarian, prostatic, and pancreatic cancers^4^. Rational results were found when PREX2 was overexpressed causing the migration and invasion capabilities to increase; while knocking down the gene resulted in decreased migration and invasion^5,6^.

Although both PREX1 and PREX2 play important roles in tumor growth, the incidence of somatic mutation in PREX2 was much higher than that of PREX1^4^. The somatic mutation of PREX2 has been reported in several cancers. Interestingly, the mutations reported for melanoma were distributed in various domains without repeating, while R155Q mutation frequency was 7.7% in breast cancer and D269H mutation frequency was 38% in squamous cell carcinoma^7^. To date, the somatic mutations of PREX2 in HCC still remain to be elucidated.

Several signal transduction pathways involved in HCC were identified including the Ras/Raf/MEK/ERK pathway, pro-angiogenic pathways, EGFR pathway, PI3K/AKT/mTOR pathway, HGF/c-Met pathway, IGF/IGFR system, and histone deacetylase inhibitors^8^. PREX2 has been identified as a PTEN inhibitor and thus plays a role in tumor proliferation^4^. The upregulation of PREX2 enhanced the proliferation and migration of HCC cells, and the mRNA expression of PREX2 in HCC tissues was upregulated compared with matched adjacent non-tumorous tissue^9^. This finding was consistent with that of our previous study, in which PREX2 accumulation led to AKT activation and enhanced proliferation^10^. However, unlike the 15 paired samples in He’s study, our 139 paired tumor and tumor adjacent specimens did not show notable alteration of PREX2 mRNA expression. Interestingly, the protein levels of PREX2 in tumor tissues were higher than those in corresponding normal tissues, which may suggest a post-translational modification^10^. Given such distinctive characteristics in HCC, we hypothesized that somatic genetic alterations may affect PI3K/AKT/mTOR pathway and we identified a mutation - S1113R on the inositol polyphosphate 4-phosphatase (IP4P) domain. Such mutation may enhance PREX2 protein stability and promote cell proliferation.

## Results

### Identification of PREX2 somatic mutation in HCC patients

**Figure 1:**
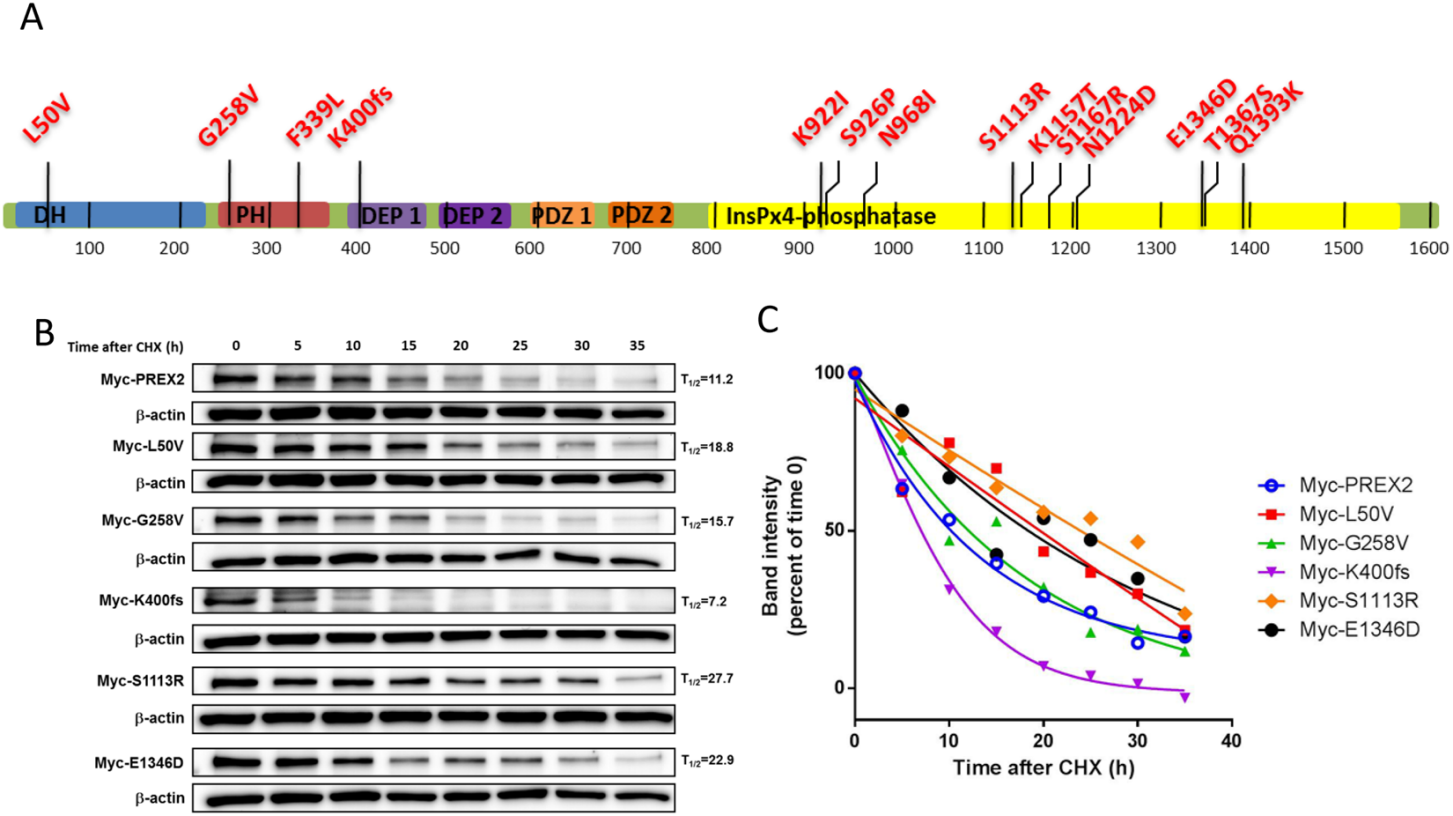
Non-silent mutations within PREX2 in HCC tumors and the protein stability analysis of wild-type and PREX2 mutants. (**A**) Non-silent somatic mutations were detected from Illumina sequencing of 68 HCC tumors. fs, frameshift deletion mutation; DH, DBL homology domain; PH, plekstrin homology domain; DEP, Dishevelled, Egl-10, and Pleckstrin domain; PDZ, post-synaptic density protein, Drosophila disc large tumor suppressor, and zonula occludens-1 protein. The C-terminal half of PREX2 displays sequence homology to an inositol phosphatase domain. (**B**) Huh7 cells expressing with wild-type PREX2 or transfected with indicated plasmids were treated with cycloheximide for the indicated hours and harvested for IB analysis. β-actin expression was used as a loading control. (**C**) Graph shows quantification of PREX2 mutants on the basis of data shown in (**B)**. Immunoblots in (**B**) are representative of at least two experiments.

PREX2 may play oncogenic roles in human cancers through mutations. Berger has reported that PREX2 somatic mutations render melanoma cells susceptible to oncogenic activities, which may perturb or inactivate one or more of its cellular functions^6^. Waddell has suggested that mutant PREX2 may be a candidate driver of pancreatic ductal adenocarcinoma^11^. To investigate whether the *PREX2* gene also harbors somatic mutations in HCC, the *PREX2* genome was analyzed in 68 pairs of hepatocellular carcinomas and peripheral blood mononuclear cells from HCC patients using HaloPlex target enrichment sequencing. The human *PREX2* genome coverage rate, mapped to the raw sequence, was 98.22%. Here, we detected 14 non-silent point mutations in *PREX2*, with 16 of 68 HCC patients harboring at least one non-silent mutation (Tables 1 and 2). Among these non-silent mutations, there were 13 non-synonymous substitutions and one frameshift truncation distributed over several domains of PREX2 (Fig. 1A and Table 1). In other words, 23.5% of HCC samples harbored at least one non-silent mutation in their *PREX2* gene. It is interesting to note that none of the somatic mutations mentioned above have been reported in the previous melanoma study^6^. Additionally, 11.8% (8/68) of HCC samples had a 1-nucleotide deletion as a frameshift mutation (K400fs) of PREX2 which resulted in a truncated form of PREX2 protein containing only the N-terminal Dbl-homologous-Pleckstrin-homology (DH-PH) tandem domain. It has been reported that the DH domain is the regulator of GEF activity and the PH domain represses the phosphatase activity of PTEN^3^. Further studies are necessary to elucidate their roles in the oncogenesis of HCC.

**Table 1:** Summary of non-silent mutations within *PREX2* in 68 human HCC tumors.

	AAC^a^	Nucleotide	Polymorphic type^b^	PBMC (n)	Tumor (n)	Sequence
Missense mutation	L50V	148 > G	SNV	0	1 of 68	CATTC[T/G]TACAC
	G258V	773 G > T	SNV	0	1 of 68	TTCTG[G/T]AAATA
	F339L	1017 T > A	SNV	0	1 of 68	TGGTT[T/A]GTTTG
	N968I	2903 A > T	SNV	0	1 of 68	GCATA[A/T]TTCTC
	K922I	2765 A > T	SNV	0	1 of 68	GGCCA[A/T]ATCTA
	S926P	2776 T > C	SNV	0	1 of 68	AAATC[T/C]CCCCA
	S1113R	3337 A > C	SNV	0	1 of 68	GCAAC[A/C]GCAAT
	K1157T	3470 A > C	SNV	0	1 of 68	GGACA[A/C]GATAC
	S1167R	3501 C > A	SNV	0	1 of 68	TTCAG[C/A]CAGGT
	N1224D	3670 A > G	SNV	0	1 of 68	CTTGG[A/G]ATCTT
	E1346D	4038 A > T	SNV	0	1 of 68	TTGGA[A/T]AAGGT
	T1367S	4099 A > T	SNV	0	1 of 68	CTCTT[A/T]CATAT
	Q1393K	4177 C > A	SNV	0	1 of 68	TTCCT[C/A]AACGG
Frameshift mutation	K400fs^c^	1200 delG	1-nucleotide Del	3 of 68	8 of 68	CGAAA[G/*]AGAAA

^a^AAC, amino acid change, ^b^SNV, single nucleotide variant; ^c^fs, frameshift mutation.

**Table 2:** Clinical characteristics of 16 HCC tumors with 14 non-silent mutations.

Sample	AAC			Age	Gender	Viral infection^a^	Tumor size(cm)	TNM stage^b^	Smoking	Drinking
1	K400fs			75	Female	NBNC	10.5	Late	No	None
2	K400fs			52	Male	HCV	2.5	Late	Yes	has, had
3	K400fs,	E1346D		79	Male	HCV	3	Late	Yes	None
4	K400fs			30	Female	HBV	13	Late	No	None
5	K400fs,	F339L,	K1157T	77	Female	HCV	6	Late	No	None
6	K400fs,	S926P		77	Male	HCV	6	Late	No	has, had
7	K400fs			58	Male	HBV	4	Late	No	None
8	K400fs			71	Female	HCV	5	Late	No	None
9	G258V			43	Male	HCV	18.5	Late	No	None
10	K922I			68	Male	HBV	12	Late	No	None
11	N968I			68	Male	HBV	10	Late	Yes	has, had
12	S1113R,	L50V		57	Male	HBV	6.5	Early	No	has, had
13	S1167R			74	Female	HBV	14	Late	No	None
14	N1224D			83	Male	HCV	11	Late	Yes	has, had
15	T1367S			49	Male	HCV	7.5	Late	Yes	has, had
16	Q1393K			53	Male	HBV	7	Late	Yes	None

^a^HBV, HBV sAg (+); HCV, anti-HCV antibody (+); NBNC, HBV sAg (−) and anti-HCV antibodies (−).

^b^According to the AJCC 7th edi Pathology staging system: Early stage, TNM stage = I; Late stage, beyond TNM stage I (TNM stage = II + IIIA +IIIB + IIIC + IV).

### Somatic mutations of PREX2 affect the half-life of proteins

In Berger’s study, such ectopic expression of mutant PREX2 worsens the progression of melanoma^6^. To examine whether these non-silent mutations play a role in oncogenesis of HCC, we constructed and transfected six PREX2 plasmids, including the wild-type, into cells separately. These five mutations were found in the first batch of 30 HCC patients, and each protein half-life was determined using cycloheximide. As expected, all mutant forms of PREX2, except for K400f, had an extended half-life compared with the wild-type PREX2 (Fig. 1B,C). A change in the reading frame may result an entirely different translation, resulting in different amino acids coded and unusually short, unusually long or even dysfunctional polypeptides. The frame shift caused by 1200 delG shortened the protein half-life. All other mutations enhanced the protein stability in various degrees, but only the half-life of S1113R was twice the length that of wild-type.

### Cancer-derived PREX2 mutant, S1113R, enhanced cell proliferation and migration

**Figure 2:**
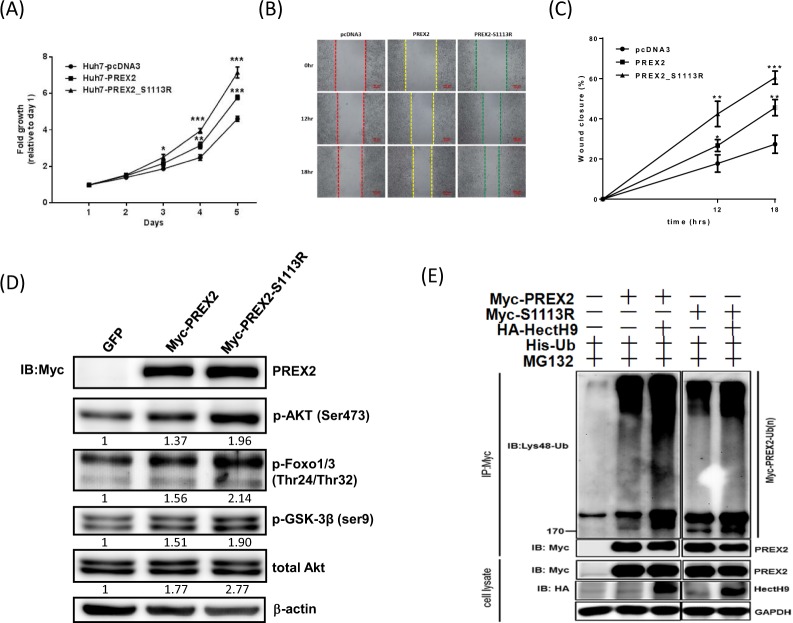
Cancer-derived PREX2 mutant, S1113R, promoted cell proliferation and migration abilities, activated AKT pathway and impaired HectH9-mediated ubiquitination. (**A**) Cell proliferation was measured in Huh7 cells transfected with indicated plasmids. (**B**) Wound-healing assay was used to evaluate the motility of Huh7 cells transfected with indicated plasmids. The percentage of wound closure was measured and compared at 12 and 18 hours later. (**C**) Graph shows quantification of wound-healing assay on the basis of data shown in (**B**). (**D**) Huh7 cells were transfected with wild-type and mutant form of PREX2 plasmids. Lysates were collected, and levels of phosphorylated AKT and downstream targets were analyzed by Western blot. β-actin was used to normalize IB data. (**E**) The *in vitro* ubiquitination assay in Huh7 cells treated with MG132 and transfected with His-Ub, along with co-transfection of Myc-PREX2, Myc-S1113R and/or HA-HectH9. Polyubiquitinated proteins were pulled down by Ni-NTA agarose and the presence of polyubiquitinated Myc-PREX2 was determined by IB with Myc antibody. Unprocessed original images of blots are shown in Supplementary Fig. S3. Data in **(A**,**C**) are presented as mean ± SD (n = 3). **p < 0.01 and ***p < 0.005, using Student’s t-test. Immunoblots in (**D**,**E**) are representative of at least two experiments.

Next, cell proliferation assays for these six cancer-derived PREX2 mutants were performed showing various effects on cell growth rates (Supplementary Fig. S1). Remarkably, PREX2-WT overexpression promoted proliferation of HCC cells and PREX2-S1113R overexpression promoted proliferation even more significantly (Fig. 2A). Then Huh7 cells were transfected to overexpress GFP, PREX2-WT or PREX2-S1113R to investigate the effects of S1113R on migration. The wound healing assay showed that Huh7^PREX2-WT^ cells and Huh7^PREX2-S1113R^ cells demonstrated quicker closure when compared with Huh7^pcDNA3^ cells (Fig. 2B,C). Taken together, we found that S1113R, a cancer-derived PREX2 mutant, significantly increased the proliferation and migration of HCC cells.

### S1113R, a cancer-derived PREX2 mutant, activates AKT pathway and impairs HectH9-mediated ubiquitination

Given that PREX2-S1113R expression in Huh7 cells conferred a strong ability to promote proliferation and migration, and some cancer mutants of PREX2 were reported to be resistant to antagonism by PTEN, a functional signaling analysis of the AKT pathway was further made^12^. In comparison to wild-type PREX2, the levels of phosphorylation of the PI3K downstream substrate AKT (S473) and those of the downstream targets of AKT of the S1113R mutant form of PREX2 were increased (Fig. 2D). The downstream targets of AKT included forkhead box protein O1 and 3 (Foxo1/3) and glycogen synthase kinase 3 beta (GSK-3*β*): and these results suggested involvement of the AKT pathway.

**Figure 3:**
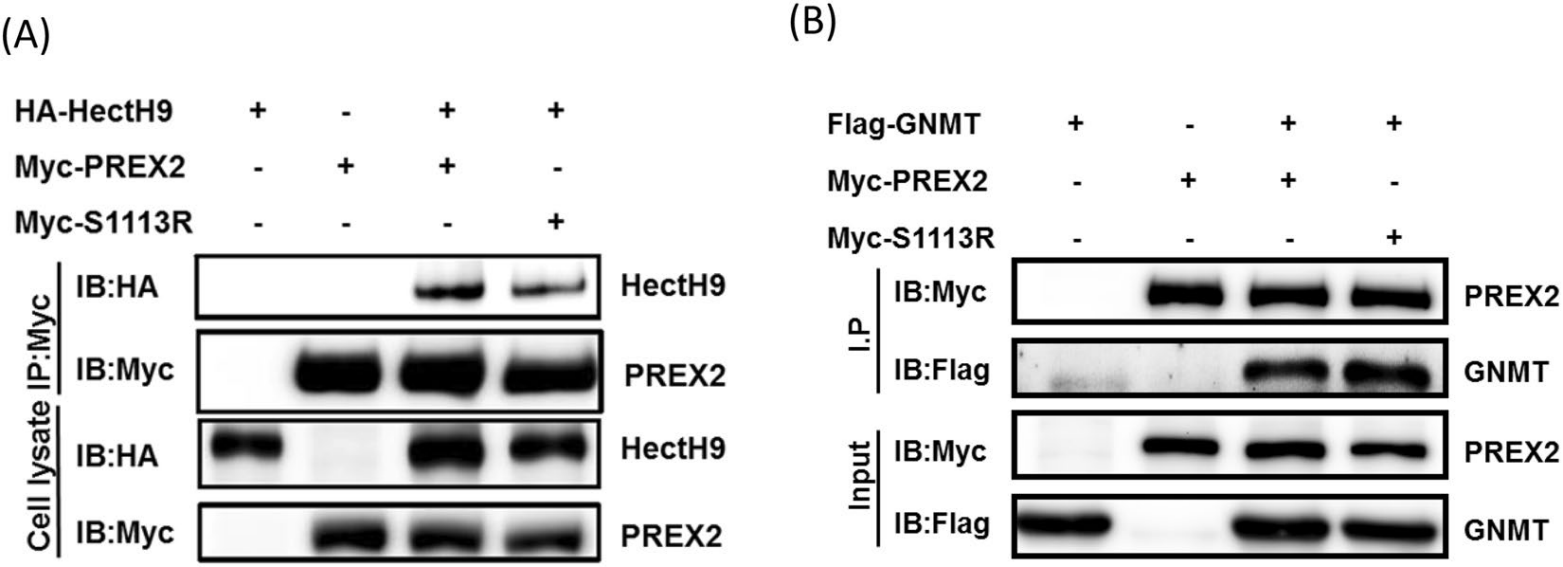
Cancer-derived PREX2 mutant, S1113R affected the interaction of HectH9, not GNMT. Huh7 cells were transfected with indicated plasmids. Lysates were collected for co-IP assay followed by IB analysis. (**A**) Levels of HectH9 and PREX2 were analyzed. (**B**) Levels of PREX2 and GNMT were analyzed. Immunoblots are representative of at least two experiments.

Previously, we examined several E3 ligases for PREX2 ubiquitination in the presence of proteasome inhibitor and identified the E3 ligase homologous to E6AP carboxyl terminus homologous protein 9 (HectH9; also known as Huwe1, Mule or ARF-BP1), which strongly promoted PREX2 ubiquitination *in vitro*^10^. HectH9 consists of a UBA, a WWE, a BH3 and a HECT domain, and its roles in cancer are controversial because HectH9 ubiquitylates p53 in some conditions, but targets the anti-apoptotic protein, myeloid cell leukemia sequence 1 (MCL1), in other conditions^13^. Through the *in vitro* ubiquitination assay, Huh7 cells treated with MG132 and transfected with His-Ub, along with co-transfection with Myc-PREX2, Myc-S1113R and/or HA-HectH9 showed various levels of ubiquitination. A reduced level of ubiquitination of Huh7 PREX2-S1113R cells was observed (Fig. 2E and Supplementary Fig. S2). In addition, S1113R affected the interaction of HectH9, not GNMT as shown in Fig. 3. This indicates that S1113R, the mutant form of PREX2, may have a way to evade HectH9-mediated degradation.

## Discussion

Here, we have demonstrated that a non-silent somatic mutation of *PREX2* is not rare in HCC (23.5%, as shown in Tables 1 and 2). In a recent melanoma study, 27% of the cohort was identified to have *PREX2* mutations^14^. In another study which recruited 100 pancreatic ductal adenocarcinoma patients, only 10% of the cohort was identified to have *PREX2* mutations^11^. Moreover, Fine and coworkers reported a map of the *PREX2* mutations associated with colorectal, kidney, lung, and pancreatic tumors^4^. Among these studies, we found only one mutation site - S926Y in lung tumor - to be the same as the one in our study, but it had a different substituent (S926P). Robinson and colleagues recruited 500 adult patients with metastatic solid tumors and found that *PREX2* was in the top four of highly mutated genes^16^. In addition, analysis of data available in public-domain databases revealed a 6.4~28.3% mutation rate of *PREX2* in HCC samples (Supplementary Table S1). Thus, *PREX2* may be a highly mutated gene in human tumors.

A review article, Srijakotre *et al*., summarized the PREX2 non-synonymous mutations in various cancers, including breast cancer, melanoma, lung cancer, pancreatic cancer, and squamous cell carcinoma^7^. The major mutations were located in the PH domain (42.9%) of lung cancer and in the C-terminal IP4P domain (64.3%) of melanoma^7^. Our HCC samples revealed that 71.4% of non-silent mutations were found within the IP4P domain (Fig. 1A). While the N-terminal DH-PH tandem domain was found to interact with the tumor suppressor PTEN resulting in mutual inhibition, this IP4P domain may also play an important role in the mutual inhibition from PTEN^4,12,15^. The PTEN tail contains a PDZ domain, which interacts with the IP4P domain (ranging from 755 to 1606) of PREX2 and is sufficient to antagonize the PREX2-driven invasion when tested in breast cancer cells, BT549 and SUM149, and in human embryonic kidney, 293 cells^12^. Also, Hodakoski and coworkers suggested that PTEN tail may inhibit PREX2 alone or work in concert with the PTEN C2 domain (interacting with the PH domain of PREX2) to further suppress PREX2 activity^15^. When subsequent examinations were applied, not all mutants located in the IP4P domain affected the PTEN binding equally. For example, both P948S and G844D mutants were from melanoma and located in the IP4P domain; the interaction between PREX2 P948S and C2-tail or full-length PTEN was in a way similar to that of wild-type PREX2, but PREX2 G844D interacted with C2-tail or full-length PTEN in a much weaker degree^12^. It is possible that S1113R, located at the IP4P domain, may affect the interaction between PTEN and PREX2; however, this interaction remains to be elucidated.

Even though both S1113R and E1346D are on the IP4P domain, only the amino acid residue of S1113 loses the capacity of being phosphorylated after the amino acid change. It has been reported that protein phosphatase 1α (PP1α) binds to the IP4P domain of PREX1 and dephosphorylates the phosphorylation of Ser1165 and thereby activates P-Rex1^17^. In Barber’s *in vitro* experiment, the S1165A mutant showed a 3-fold increase compared with the basal P-Rex1 Rac-GEF activity^17^. Thus, it is rational to speculate that S1113R may have noteworthy effects on PREX2. Indeed, when examining the protein half-life of various non-silent mutations, S1113R’s was markedly extended. Such mutant activity also promoted proliferation and migration abilities of PREX2 in addition to activating AKT pathway and impairing HectH9-mediated ubiquitination. Previously, we characterized a tripartite relationship of GNMT-HectH9-PREX2 in the pathogenesis of HCC. We reported that GNMT interacted with the paired PDZ domain of PREX2 and promoted the lysine 48-linked ubiquitination of PREX2^10^. In other words, since S1113R is located in the IP4P domain, the point mutation may not affect the interaction between PREX2 and GNMT, but may possibly affect the one between PREX2 and HectH9 (Fig. 3). Thus, S1113R may be a gain-of-function mutant related to the malignancy of tumors and may be used as a predication marker for diagnosis.

One more interesting observation in the present study was that the K400f, a frameshift deletion mutation, frequently showed up not only in HCC patients’ PBMC samples (3/68) but also in tumor samples (8/68). Such *PREX2* mutation may result in a truncated form of PREX2 having only a DH-PH tandem domain. Since the DH domain is the main area regulating catalytic GEF activity whereas the PH domain is the regulator of inhibition of PTEN phosphatase activity, it was expected to have functional impact^15,18^. When the clinical characteristics among HCC patients were analyzed, the viral infection stood out from other factors including tumor size, tumor type (solitary/multiple), TNM stage (early/late), smoking history, cirrhosis and invasion conditions (Supplementary Table S2). Five out of eight patients with K400f mutation are anti-HCV Ab-positive and such results suggested K400f may be related to the oncogenic role of hepatitis C virus.

## Materials and Methods

### Hepatocellular Carcinoma (HCC) patients

All the HCC specimens were excised liver tumors obtained from the Taiwan Liver Cancer Network (TLCN). Informed consent was obtained from all patients before surgery. Additionally, clinical and pathological data were provided by TLCN. For the analysis of somatic mutations in PREX2, DNA of specimens from 68 HCC patients (mean age, 59.7 ± 13.4) and matched peripheral blood samples were used. We divided them into 3 groups according to types of hepatitis viral infection: 39 patients (25 males and 14 females) were HBsAg-positive, 22 patients (14 males and 8 females) were anti-HCV Ab-positive, and 7 patients (3 males and 4 females) were hepatitis B and C negative. This study was approved for de-identified data analysis for research purposes by the Institutional Review Board of National Yang Ming University and the user committee of TLCN. All experiments were performed in accordance with relevant guidelines and regulations.

### Plasmids and constructs

The pGNMT-Flag construct has been described previously^19^. To generate the lentiviral expression construct for GNMT, the coding sequences for Flag-tagged GNMT were inserted into the lentiviral vector pLKO-AS3w.hyg (National RNAi Core Facility, Academia Sinica, Taipei, Taiwan). For the overexpression experiments (pLKO-AS3w.eGFP.puro), the packaging plasmid (pCMV-ΔR8.91), and the envelope plasmid (pMD.G) were also obtained from the National RNAi Core Facility. Myc-PREX2 construct was obtained from Dr. H.C.E. Welch. (His)_6_-ubiquitin was a gift from Dr. P. H. Tseng. (His)_6_-ubiquitin and HA-HectH9 have been described previously^20^. The mutants (L50V, G258V, K400f, S1113R, and E1346D) were generated by site-directed mutagenesis using Myc-PREX2 as the template (Phusion SDM kit, Thermo Scientific). The primer sequences are listed in Supplementary Table S3.

### Cell cultures and transfection

Huh7 cells were cultured in complete Dulbecco’s modified Eagle’s medium (DMEM) (DMEM containing 10% heat-inactivated fetal bovine serum, penicillin (100 U/ml), streptomycin (100 μg/ml), nonessential amino acids (0.1 mM), and L-glutamine (2 mM) (Gibco [Thermo Fisher Scientific, Waltham, MA, USA]). Lentivirus-infected cells were grown in complete DMEM supplemented with either puromycin (1μg/ml, Sigma-Aldrich, St. Louis, MO, USA) alone or puromycin (1 μg/ml) and hygromycin (100 μg/ml, Invitrogen [Thermo Fisher Scientific]) together. Plasmid DNA was transfected by using TurboFect™ Reagent (Fermentas, Hanover, MD, USA). All transfections were performed according to the manufacturers’ instructions.

### Antibodies

The following antibodies were used at dilutions recommended by manufacturers. Mouse GNMT (Glycine N-methyltransferase) monoclonal primary antibody (M003) was purchased from YMAC Biotech Co. Mouse Myc monoclonal primary antibody (46–0603) was purchased from Invitrogen. Mouse HA monoclonal primary antibody (MMS-101R) was purchased from Covance. Mouse monoclonal primary antibodies of β-actin (A5441) and FLAG (F1804), rabbit GAPDH monoclonal primary antibody (G8795), and rabbit PREX2 polyclonal antibody (HPA015234) were purchased from Sigma. Rabbit ubiquitin, K48-specific monoclonal primary antibody (05–1307) was purchased from Millipore. Rabbit monoclonal primary antibodies of total AKT (4691S), phospho-AKT (S473) (4060S), and phospho-GSK3b (S9) (5558S) were purchased from Cell Signaling Technology. Rabbit phospho-Foxo1 (T24)/Foxo3a (T32) polyclonal primary antibody (9464S) was also purchased from Cell Signaling Technology.

### Viral infection

For lentivirus preparation, Huh7 cells were cotransfected with a packaging plasmid-pCMV-ΔR8.91, a VSV-G envelope expressing plasmid-pMD.G and the lentiviral constructs by using TurboFect™ Reagent (Fermentas). The packaging plasmid (pCMV-DR8.91) and the envelope plasmid (pMD.G) were obtained from the National RNAi Core Facility.

### Immunoprecipitation and Immunoblotting

Immunoprecipitation (IP) and immunoblotting (IB) were performed essentially as previously described with some modifications^21,22^. In brief, cultured cells were lysed by E1A lysis buffer (250 mM NaCl, 50 mM HEPES (pH 7.5), 0.1% NP40 and 5 mM EDTA) supplemented with a protease inhibitor cocktail (Roche, Mannheim, Germany) and phosphatase inhibitors (1 mM NaF, 5 mM NaPPi, and 10 mM Na_3_VO_4_).The concentrations of the cell lysates were measured using Bio-Rad protein assay (Bio-Rad, Hercules, MA, USA). The lysates were incubated with antibodies either overnight or for 4 h at 4 °C and followed by incubation with protein-A/G sepharose (GE Healthcare, Pittsburgh, PA, USA) for 2 h. The beads were washed with lysis buffer and eluted in sample buffers for SDS-PAGE and Western blot analyses.

### Cycloheximide analysis

For cycloheximide (CHX) treatment, Huh7 cells were transfected with indicated plasmids for 48 h and then treated with CHX (70 μg/ml, sigma) for indicated hours, harvested and subjected to SDS-PAGE and Western blot analyses. Densitometry was performed using Dolphin View Band Tool (Wealtec Corp., Sparks, NV).

### The *in vitro* ubiquitination assay

The *in vitro* ubiquitination assay was performed as described elsewhere^12^. In brief, Huh7 cells were transfected with the indicated plasmids for 36–48 h, treated with 10 μM MG132 (Calbiochem) for 4 h, and lysed with denaturing buffer (6 M guanidine-HCl, 0.1 M Na_2_HPO_4_/NaH_2_PO_4_, 10 mM imidazole). The cell extracts were then incubated with Ni-NTA agarose for 3 h, rinsed, and subjected to Western blot analysis.

### Cell proliferation assay

Cells were seeded in a 96-well plate (1,000 cells per well) in at least triplicate for each experiment. At every indicated time point, 10 uL of alamarBlue® (10%, Invitrogen [Thermo Fisher Scientific]) was added and further incubated for 4 h at 37 °C. Fluorescence of the reduced alamarBlue® was measured by microplate reader (Synergy HT, BioTek Instruments, Winooski, VA, USA).The amount of fluorescence for each group was normalized the day after seeding (day1) and shown as fold increase. All data are presented as mean ± SD.

### Wound healing assay

Cell culture migration insert (ibidi) was placed into a 24-well plate. Cells were trypsinized and resuspended in complete culture medium and were placed into each side of the insert. The inserts were removed at indicated time points before pictures were taken.

### HaloPlex target enrichment sequencing

All DNA samples were evaluated for several quality criteria prior to Illumina sequencing. DNA concentration was measured using Epoch system (BioTek) and DNA amounts more than 500 ng were taken into consideration. In addition, structural integrity of DNA was checked by gel electrophoresis, and degraded samples were removed from consideration. The HaloPlex target enrichment system (Agilent Technologies, Santa Clara, CA, USA) was used to capture and determine the PREX2 genomic DNA sequence by the specifically designed probes as recommended by the manufacturer. The genomic DNA (200–250 ng) from HCC specimens were fragmented by restriction enzyme digestion and circularized by hybridization to probes whose ends are complementary to the target fragments. The probe contained a method-specific sequencing motif that was incorporated during the circularization. The circular molecules were then closed by ligation and target DNA were captured using Streptavidin beads. Amplified circular DNA targets were enriched and subjected to sequencing.

### Sequence data processing and identification of somatic mutation

Adapter sequence trimming, quality filtering, coverage determination and the initial assembly of sequence contigs were performed using GEMINI mainframe program and CLC Genomics Workbench^23^. Candidate single nucleotide polymorphism (SNP) and insertion-deletion (indel) mutations were identified by comparison of read realignment with Single Nucleotide Polymorphism database (dbSNP). To identify somatic substitutions and indels, we compared the realignment from HCC tumor and matched germline DNA with a reference genome (Ref_NCBI_GRCh37_hg19) by using GEMINI, and known SNPs present in the dbSNP database were filtered out.

### Statistical analysis

Statistical analysis was performed using SPSS software (version 13, SPSS Inc) and *p* < 0.05 was considered to be statistically significant. Pearson χ^2^ or Fisher’s exact tests were used to evaluate the association between PREX2 expression and different clinicopathological characteristics of HCC patients. Multivariate logistic regression models were used to adjust for covariate effects on the odds ratio. Comparisons between groups were made using the Student’s t-test. The Kaplan-Meier estimation method was used for overall survival analysis, and a log-rank test was used to compare differences. Multivariate survival analyses were conducted using a Cox proportional hazards regression model.

## Electronic supplementary material

Supplementary Information for SR_Figures and Tables
