# Supplementary material for: Somatic mutations of PREX2 gene in patients with hepatocellular carcinoma
Source: Sci Rep. 2019 Feb 22;9:2552. doi: 10.1038/s41598-018-36810-5 (PMC6385191; doi:10.1038/s41598-018-36810-5)
Supplement: Supplementary file 1 — Supplementary Information for SR_Figures and Tables [file 41598_2018_36810_MOESM1_ESM.docx]

**Supplementary Information**

**Somatic mutations of *PREX2* gene in patients with hepatocellular carcinoma**

Ming-Hui Yang^1^, Chia-Hung Yen^1,2^, Yen-Fu Chen^1^, Cheng-Chieh Fang^1^, Chung-Hsien Li^1^, Kuo-Jui Lee^1^, Yi-Hsiung Lin^1^, Chien-Hui Weng^1^, Tze-Tze Liu^3^, Shiu-Feng Huang^4^, Bin Tean Teh^5^ and Yi-Ming Arthur Chen ^1,6,7†^

^†^**Corresponding author:**

Professor Yi-Ming Arthur Chen, M.D., Sc.D.

Address: No. 100, Shih-Chuan Ist Rd, Kaohsiung City, Taiwan 80708

Phone number: 886-7-3121101

Fax number: 886-7-3222783

Email address: [arthur@kmu.edu.tw](mailto:arthur@kmu.edu.tw)

Table of Content:

1. Supplementary Figures 1-2.
2. Full unedited images for Figures 1B, 2D-E and 3A-B
3. Supplementary Tables 1-3


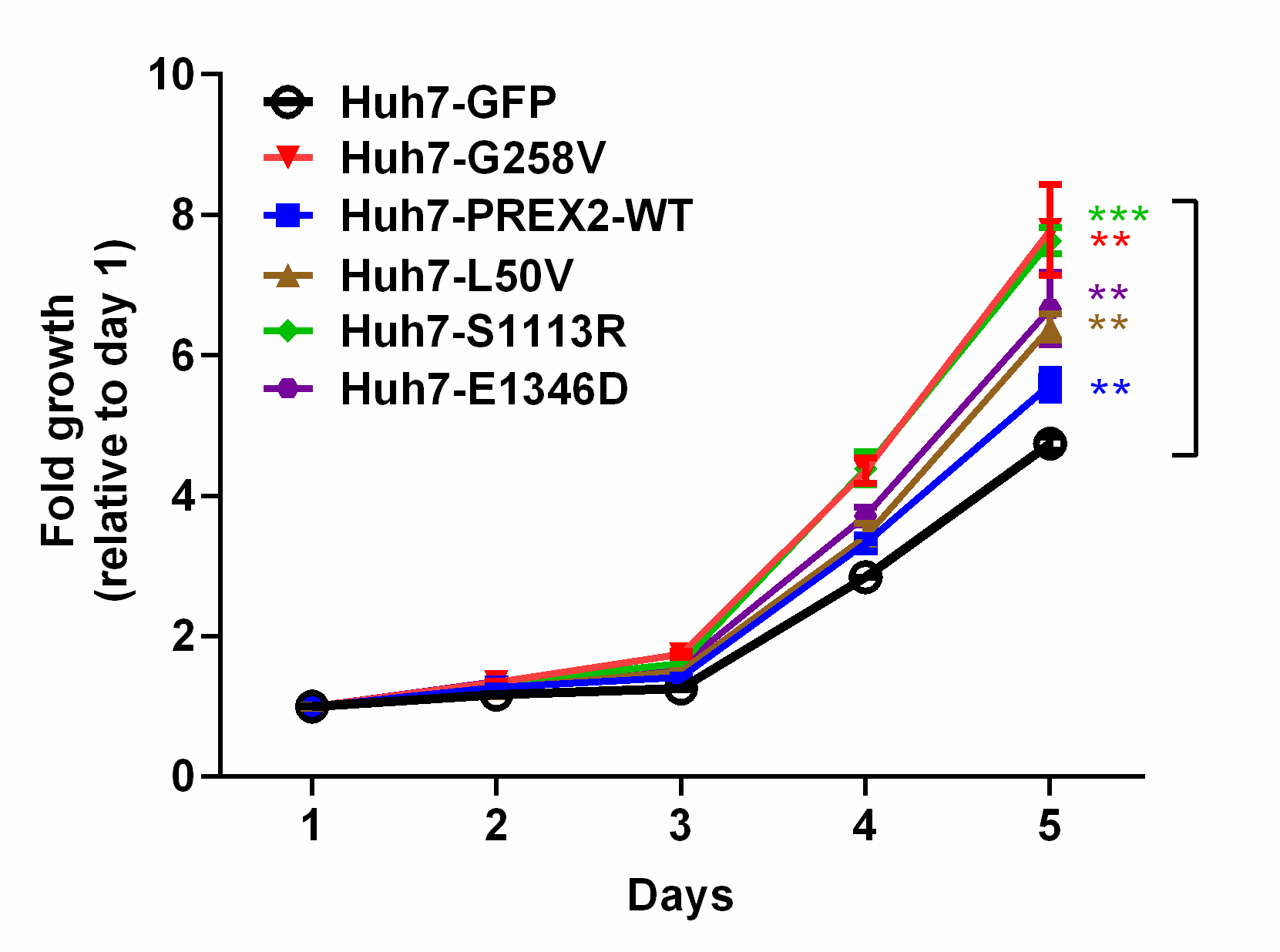


**Figure S1**. Cancer-derived PREX2 mutants affect cell proliferation differently. Cell proliferation was measured in Huh7 cells transfected with indicated plasmids. Experimental results are presented as mean ± SD. **p <0.01, using Student’s t-test.

**
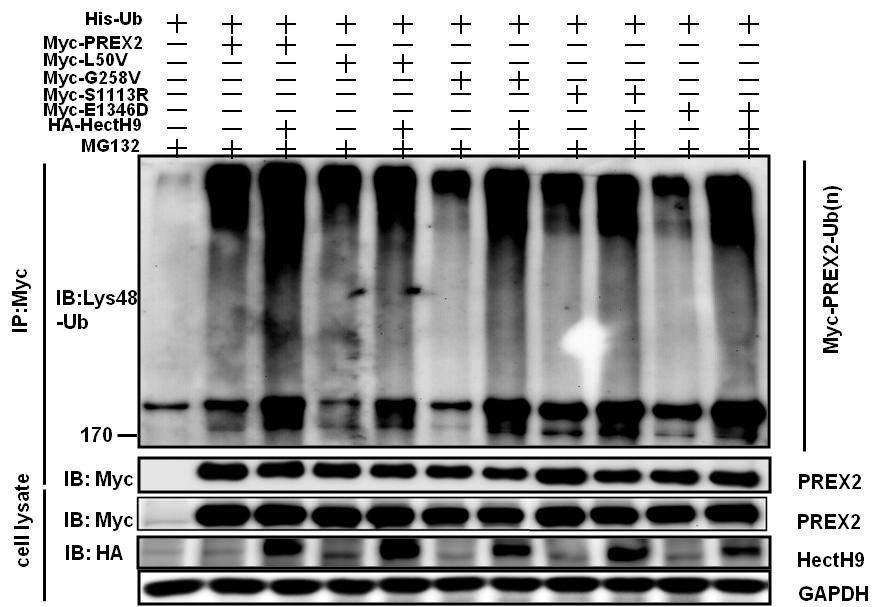
**

**Figure S2.** Mutations of PREX2 impaired HectH9-mediated ubiquitination. The *in vivo* ubiquitination assay shows Huh7 cells which have been treated with MG132 and transfected with His-Ub, along with co-transfection of Myc-PREX2, Myc-L50V, Myc-G258V, Myc-S1113R, Myc-E1346D and/or HA-HectH9. Polyubiquitinated proteins were pulled down by Ni-NTA agarose and the presence of polyubiquitinated Myc-PREX2 was determined by IB with Myc antibody. Immunoblots are representative of at least two experiments.

Full unedited images for Figures 1B and 2D-E

Full unedited images for Figure 3

Fig 3B

Fig 3A

Supplementary Table S1 PREX2 somatic mutation rates of HCC patients in various countries.

| Primary site | Country | N | PREX2 (%) |
| --- | --- | --- | --- |
| Liver | Japan | 244 | 28.3 |
|  | United States | 188 | 6.4 |
|  | France | 234 | 7.3 |

Supplementary Table S2 Mutations of PREX2 from 68 HCC patients and the associations with their clinical characteristics.

| Characteristics | With mutation | Without mutation | All | *P* |
| --- | --- | --- | --- | --- |
| **Cirrhosis** |  |  |  | 0.51 |
| Negative | 9 | 34 | 43 |  |
| Positive | 7 | 18 | 25 |  |
| **Viral infection**^a^ |  |  |  | <0.05 |
| NBNC | 1 | 6 | 7 |  |
| HBV | 7 | 32 | 39 |  |
| HCV | 8 | 14 | 22 |  |
| **Tumor size (cm)** |  |  |  | 0.11 |
| <5 | 3 | 21 | 24 |  |
| ≥5 | 13 | 31 | 44 |  |
| **Smoking** |  |  |  | 0.69 |
| (+) | 10 | 29 | 39 |  |
| (-) | 6 | 22 | 28 |  |
| **TNM**^b^ **stage** |  |  |  | 0.44 |
| Early | 2 | 11 | 13 |  |
| Late | 14 | 41 | 55 |  |
| **Tumor type** |  |  |  | 0.24 |
| Solitary | 3 | 21 | 24 |  |
| Multiple | 13 | 31 | 44 |  |
| **Vascular invasion** |  |  |  | 0.51 |
| Absent | 3 | 14 | 17 |  |
| Vein invasion or vein  tumor thrombosis | 13 | 38 | 51 |  |

^a^HBV, HBV sAg (+); HCV, anti-HCV antibody (+); NBNC, HBV sAg (−) and anti-HCV antibodies (−).

^b^Early stage, TNM stage = I; Late stage, beyond TNM stage I (TNM stage = II+IIIA+IIIB+IIIC+IV).

Supplementary Table S3. Summary of primers used in site-directed mutagenesis to generate *PREX2* mutants.

|  | |
| --- | --- |
| Target | Primer |
| L50V-anti | 5'-ACT GGT TCA TTC TGT GTA CGA ATG-3‘ |
| L50V | 5'-TGT TTC ATG CAG GCA TTC GTA CAC-3 |
| G258V-anti | 5‘-ACC CGT TCT TGA ATA TTT ACA GAA-3‘ |
| G258V | 5'-TAC TGA AAA TTT CTT CTG TAA ATA-3' |
| K400-anti | 5'-GAA CGT AGT CAG TTT TCT TTT CG-3‘ |
| K400 | 5‘-TCT GAT CAA AGA CCG AAA AGA AA-3‘ |
| S1113R-anti | 5'-CGA TGG AAT TCC TAT TGC GGT TGC-3‘ |
| S1113R | 5'-GTT TTC AGT GAC TGC AAC CGC AAT-3' |
| E1346D-anti | 5‘-AAA GTA AAA GGA AAC CTT ATC CAA-3‘ |
| E1346D | 5‘-TGC ACT ATT TGA TTT GGA TAA GGT-3' |
